# Exploring Frailty Status and Blood Biomarkers: A Multidimensional Approach to Alzheimer’s Diagnosis

**DOI:** 10.3390/geriatrics10050133

**Published:** 2025-10-17

**Authors:** Aurora Cermelli, Armando Crisafi, Alberto Mario Chiarandon, Giorgia Mirabelli, Chiara Lombardo, Virginia Batti, Silvia Boschi, Elisa Maria Piella, Fausto Roveta, Innocenzo Rainero, Elisa Rubino

**Affiliations:** 1 Aging Brain and Memory Clinic, Department of Neuroscience “Rita Levi Montalcini”, University of Torino, 10126 Turin, Italy; aurora.cermelli@unito.it (A.C.); alberto.chiarandon@unito.it (A.M.C.); giorgia.mirabelli468@edu.unito.it (G.M.); c.lombardo@unito.it (C.L.); virginiabatti@gmail.com (V.B.); silvia.boschi@unito.it (S.B.); elisamaria.piella@unito.it (E.M.P.); fausto.roveta@unito.it (F.R.); innocenzo.rainero@unito.it (I.R.); 2 Department of Clinical and Experimental Medicine, Internal Medicine, Garibaldi Hospital, University of Catania, Via Palermo 636, 95122 Catania, Italy; armando.crisafi95@gmail.com; 3 Center for Cognitive Disorders and Dementias (CDCD), Department of Neuroscience and Mental Health, AOU Città della Salute e della Scienza di Torino, 10126 Turin, Italy

**Keywords:** Alzheimer’s disease, frailty, plasma biomarkers, early diagnosis, MPI

## Abstract

**Background:** Frailty is a multidimensional syndrome reflecting reduced physiological reserve, increasingly recognized as a relevant factor in the clinical assessment of older adults with cognitive disorders. **Objective:** To explore the association between frailty, as measured by the Multidimensional Prognostic Index (MPI), cognitive performance, and plasma biomarkers of Alzheimer’s disease (AD), and to examine the correlation between plasma and cerebrospinal fluid (CSF) biomarkers. **Methods:** This cross-sectional observational study included 40 patients (mean age 68.0 ± 9.0 years; 42.5% female) undergoing a diagnostic workup for cognitive decline. Patients were classified into AD (n = 20) and non-AD (n = 20) groups based on CSF AT[N] profiles. Frailty was assessed using the MPI. Linear and logistic regression models adjusted for age, sex, and education examined associations between MPI, cognitive scores, and plasma biomarkers (Aβ42, Aβ42/40, p-tau181, NfL). Correlations between plasma and CSF biomarkers and ROC analyses were also performed. **Results:** The AD group showed significantly higher plasma p-tau181 levels and MPI scores. MPI was positively associated with plasma p-tau181 levels (β = 4.26, *p* = 0.009). Plasma p-tau181 correlated strongly with CSF p-tau181 (R = 0.523, *p* < 0.001) and with CSF Aβ42/40 ratio (R = −0.541, *p* < 0.001) and showed high diagnostic accuracy (AUC = 0.910). Combining MPI with plasma biomarkers improved classification between AD and non-AD cases (AUC = 0.941). **Conclusions:** These findings support the value of incorporating frailty assessment in the diagnostic process of AD. The integration of geriatric tools and blood-based biomarkers may improve early detection and promote a more comprehensive approach in dementia evaluation.

## 1. Introduction

In recent years, frailty has emerged as a key factor in the assessment of older adults with neurocognitive disorders [1]. Frailty is not simply a clinical descriptor of aging but a multidimensional syndrome that encompasses the cumulative decline of physiological systems and an increased susceptibility to stressors [2,3]. This vulnerability can significantly alter disease presentation, mask underlying pathology, or amplify functional impact in the context of dementia [4].

Several biological mechanisms proposed to underlie frailty—such as chronic low-grade inflammation, immune dysregulation, mitochondrial dysfunction, and oxidative stress—have also been investigating in neurodegeneration [5,6]. These overlapping pathways suggest a possible shared substrate between physical frailty and Alzheimer’s disease (AD), supporting the idea that frailty may not only co-exist with cognitive decline but may actively influence its trajectory [7,8].

Recent studies suggest that frailty may have specific neurobiological bases [9,10]. Neuroimaging data show that frailty is associated with reduced brain volume, particularly in the frontal and temporal cortices and in the thalamus, which are regions involved in higher-level cognitive functions. Other techniques, like diffusion tensor imaging, have also shown that frail individuals tend to have lower integrity in some white matter tracts, such as the corpus callosum and superior longitudinal fasciculus. These brain changes could reflect the long-term impact of physiological stress on the central nervous system, which reduces its ability to adapt and recover. Interestingly, cognitively frail individuals often perform similarly to those with mild cognitive impairment on neuropsychological tests, but their brain structure and function may not show the typical patterns seen in AD. For example, they usually do not present hippocampal atrophy or the specific neurophysiological alterations seen in early AD. Overall, these findings suggest that frailty affects both brain and body, with biological mechanisms that partly overlap with neurodegeneration but also remain distinct [11].

AD is a progressive neurodegenerative disorder and the leading cause of dementia globally [12]. Early detection is essential, particularly considering recent advances in disease-modifying therapies that appear most effective when administered at initial stages [13,14]. However, conventional diagnostic approaches, such as cerebrospinal fluid (CSF) analysis, are invasive and not always feasible for widespread use. As a result, there is growing interest in identifying non-invasive biomarkers, especially those detectable in blood, as a more practical and accessible alternative to CSF testing. Recent studies have highlighted the potential of specific plasma biomarkers—including amyloid beta (Aβ42, Aβ42/40), phosphorylated-tau proteins (p-tau181, p-tau217, p-tau231), and neurofilament light chain (NfL), all of which are closely associated with AD-related neuropathological changes [15].

Frailty has also emerged as a relevant factor influencing both the onset and progression of neurocognitive disorders [16] and has shown predictive value for cognitive decline and adverse outcomes in older adults [17,18]. Considering this, incorporating frailty measures into dementia research may yield more ecologically valid and clinically meaningful results. Tools like the Multidimensional Prognostic Index (MPI), derived from the Comprehensive Geriatric Assessment, enable clinicians to capture aspects of vulnerability beyond traditional diagnostic frameworks, reflecting real-world complexity in aging populations [19,20]. By combining information on comorbidities, cognitive and functional status, nutrition, medications, and social context, the MPI supports a more holistic and prognostically informative evaluation—essential in tailoring management strategies for older patients with suspected AD.

While several studies suggest that frailty may amplify the severity of dementia, including AD, its relationship with AD-specific biomarkers remains poorly characterized. Some evidence links frailty with worse cognitive performance, but the underlying biological correlates are not yet well defined [21,22,23]. At the same time, it is important to recognize that frailty, as captured by the MPI, reflects a broad multidomain vulnerability and may not directly correspond to the pathological signatures of AD biomarkers which capture more disease-specific changes. It should be underlined that our study does not directly test whether frailty modulates neurodegeneration but rather explores its association with cognitive status and biomarker profiles.

In this context, the Finnish Geriatric Intervention Study to Prevent Cognitive Impairment and Disability (FINGER) showed that a multidomain lifestyle-based intervention including diet, exercise, cognitive training, and vascular risk monitoring can help slow cognitive decline in at-risk older adults, supporting the potential of targeting frailty in dementia prevention [24].

The aim of this study is to investigate the association between frailty, as measured by MPI, and both cognitive function and plasma biomarkers in individuals with cognitive impairment. Given the increasing recognition of frailty as a multidimensional clinical condition with prognostic value in older adults, this study aims to evaluate how functional vulnerability contributes to Alzheimer’s disease diagnosis. In addition, we investigated the correlation between plasma levels of key biomarkers (Aβ42, Aβ40, p-tau181 and NfL) and their corresponding concentrations in CSF.

Overall, this research aims to integrate two complementary domains—biomarker-based diagnostics and frailty assessment—with the broader goal of improving early detection and clinical management of Alzheimer’s disease. This combination may offer a useful contribution to the diagnostic process of neurocognitive disorders.

## 2. Materials and Methods

### 2.1. Study Design

This study was conducted in the Memory Clinic at the Città della Salute e della Scienza University Hospital in Turin. Data collection was carried out between February 2024 and November 2024.

Participants were enrolled in a dedicated clinical evaluation program, where they underwent all procedures as required by the study protocol during two separate visits. On the first visit, demographic, and medical history information, including risk factors, pharmacotherapy, and comorbidities, were collected. Participants were required to have a family member present for accurate data gathering. Following this, a neurological exam and a comprehensive neuropsychological assessment were conducted. On the second day, blood samples were collected, and a lumbar puncture was performed for cerebrospinal fluid (CSF) collection. Additionally, frailty and functional autonomy were assessed through the MPI and the Activities of Daily Living (ADL) and Instrumental Activities of Daily Living (IADL) scales, which were administered to the caregivers.

### 2.2. Participants and Exclusion Criteria

Only patients with a neurocognitive disorder were included in the study and were subsequently classified into two groups: those with AD and those without AD (non-AD). The non-AD group included subjects with neurodegenerative conditions that did not meet the diagnostic criteria for AD, such as frontotemporal dementia (FTD), dementia with Lewy bodies (DLB), vascular dementia (VD), mixed forms, and cases with unspecified origin. The diagnosis of AD followed the diagnostic criteria outlined by the National Institute on Aging and the Alzheimer’s Association (NIA-AA) in 2018 [13,15], using the AT[N] system to identify patients within the Alzheimer’s disease continuum (criteria A and T). The diagnoses of FTD, DLB, VD, and other neurocognitive disorders were established according to internationally recognized diagnostic criteria specific to each condition [25,26,27,28]. Subjects whose cognitive decline could be attributed to non-neurodegenerative causes, as determined by MRI analysis, were excluded.

### 2.3. Biological Samples Collection

CSF and blood samples were collected on the same day following an overnight fast, processed, and stored according to standardized protocols. CSF was obtained by lumbar puncture using standard procedures [29,30], collected in polypropylene tubes, centrifuged within 1 h at 2000× *g* for 10 min at 4 °C, aliquoted, and stored at −80 °C. Blood was drawn into EDTA tubes, centrifuged at room temperature within 1 h at 2000× *g* for 10 min, aliquoted, and stored at −80 °C. Concentrations of Aβ40, Aβ42, p-tau181, and total tau (t-tau) in both CSF and plasma were measured using the Lumipulse G1200II platform with commercially available kits (Fujirebio Europe, Ghent, Belgium), based on a two-step sandwich chemiluminescent enzyme immunoassay (CLEIA). Neurofilament light levels were also assessed using the same platform. A quote of the participants included in this study were also part of the cohort previously analyzed [31].

### 2.4. Neuropsychological Assessment

The neuropsychological assessment consists of a clinical interview, followed by the administration of standardized tests to evaluate global cognitive function and specific cognitive domains. The tests include the Mini-Mental State Examination (MMSE) [32], Montreal Cognitive Assessment (MoCA) [33], Clock Drawing Test (CDT) [34], and Frontal Assessment Battery (FAB) [35]. The results from these tests are adjusted and compared with normative data based on the patient’s age, education level, and gender, allowing for a structured and objective evaluation.

### 2.5. Frailty Assessment

Frailty was assessed using a biopsychosocial approach with the MPI, which includes standardized scales to evaluate functional autonomy through ADL and IADL scales [36,37]. The MPI also includes assessments of cognitive function (Short Portable Mental Status Questionnaire [SPMSQ] [38]), risk of pressure sores (Exton Smith Scale [ESS] [39]), motor function and nutritional status (Mini Nutritional Assessment [MNA] short form [40]), comorbidities (Cumulative Illness Rating Scale [CIRS] [41]), and the number of medications taken by the patient. The final MPI score ranges from 0 to 1, categorizing the patient’s risk of mortality as low (≤0.33), moderate (0.34–0.66), or high (>0.66) [42]. The caregiver’s input is essential for gathering accurate information regarding the patient’s difficulties in performing daily tasks.

### 2.6. Statistical Analysis

Data were expressed as mean standard deviation for continuous variables, as median and interquartile range for ordinal variables, and as count and percentage for categorical variables. The normality of distribution was assessed using the Shapiro–Wilk test. Group comparisons between patients with AD and non-AD were carried out using parametric or non-parametric tests, depending on the nature and distribution of the variables. Specifically, Welch’s *t*-test was used for continuous variables with normal distribution, while the Mann–Whitney U test was applied for non-normally distributed data. For categorical variables, Chi-squared or Welch-corrected tests were used as appropriate.

Pearson’s correlation was used to examine associations between CSF and plasma biomarkers, and to explore the relationship between the MPI and biomarkers. Additional exploratory correlation analyses were conducted between plasma biomarkers and individual functional, cognitive physical, and frailty-related variables. These analyses were repeated separately within AD and non-AD subgroups.

Linear regressions were used to assess the association between plasma p-tau181 and selected cognitive and functional variables (IADL and SPMSQ), adjusting for age, sex, and education. Logistic regression models were developed with AD diagnosis as the dependent variable and plasma biomarkers and MPI as predictors. Exploratory logistic regression models including an interaction term between plasma p-tau181 and MPI were also tested to assess potential moderation effects. Receiver Operating Characteristic (ROC) analyses were conducted to assess the diagnostic accuracy of plasma biomarkers, alone and in combination with MPI, in discriminating AD from non-AD.

Cut-off values for plasma biomarkers were estimated based on the highest Youden Index (sensitivity + specificity − 1). A *p*-value < 0.05 was considered statistically significant. All statistical analyses were performed using R version 4.2.3 (The R Project for Statistical Computing, Vienna, Austria) and Jamovi version 2.3.28 (The Jamovi Project).

## 3. Results

### 3.1. Cohort Description

We finally enrolled a total of 40 subjects (20 AD and 20 non-AD). The mean age was 66.2 ± 9.6 years in the non-AD group and 69.8 ± 8.2 in the AD group. The average MMSE score was 25.7 ± 4.7 in the non-AD group and 21.6 ± 7.6 in the AD group, while the median MPI score was 0.188 [0.062–0.203] and 0.250 [0.188–0.328], respectively. According to the MPI classification (low, medium, high risk), in the non-AD group 18 (90%) patients were classified as low risk, 2 (10%) as medium risk, and none as high risk. In the AD group, 15 (75%) patients were classified as low risk, 5 (25%) as medium risk, and none as high risk.

Full descriptive characteristics are reported in Table 1. No significant differences were found between groups in age, sex, or education. As expected, significant group differences emerged in key CSF and plasma biomarkers, as well as in global cognition and MPI scores. Full descriptive statistics are reported in Table 1.

### 3.2. Associations Between Plasma Biomarkers, Frailty, and Clinical Variables

Plasma p-tau181 showed a strong negative correlation with the CSF Aβ42/40 ratio (R = −0.541, *p* < 0.001) and a strong positive correlation with CSF p-tau181 (R = 0.523, *p* < 0.001). Similarly, the plasma Aβ42/40 ratio was strongly correlated with its CSF counterpart (R = 0.658, *p* < 0.001) and negatively correlated with CSF p-tau181 (R = −0.321, *p* < 0.05). When frailty was taken into account, a moderate positive correlation emerged between MPI and plasma p-tau181 levels (R = 0.452, *p* < 0.05).

In the first model, higher levels of plasma p-tau181 were significantly associated with increased odds of AD diagnosis (β = 4.89, *p* = 0.004). MPI showed a trend toward significance (β = 6.24, *p* = 0.070). In a subsequent model including the interaction term, both plasma p-tau181 (β = 10.78, *p* = 0.033) and MPI were positively associated with diagnosis.

Considering all our sample, MPI was significantly associated with higher plasma p-tau181 levels (β = 4.26, *p* = 0.0093). Additional exploratory correlation analyses were conducted between plasma biomarkers and individual functional, cognitive, physical and frailty-related variables, using linear regression models adjusted for age, sex, and education. In the full sample, higher p-tau181 levels were significantly associated with poorer instrumental functioning (IADL; β = −0.225, *p* = 0.005) and worse cognitive performance (SPMSQ; β = 0.255, *p* = 0.001). To investigate group-specific effects, we also ran stratified correlation analyses by diagnostic group, which revealed that these associations were predominantly driven by the AD subgroup. In AD patients, plasma p-tau181 levels were significantly correlated with both IADL (R = −0.41, *p* = 0.043) and SPMSQ (R = 0.46, *p* = 0.032), while no such associations were observed in the non-AD group (R = −0.16, *p* = 0.501; R = −0.04, *p* = 0.882, respectively). Across all models, none of the covariates (age, sex, education) showed significant effects.

### 3.3. Diagnostic Performance

**Figure 1:**
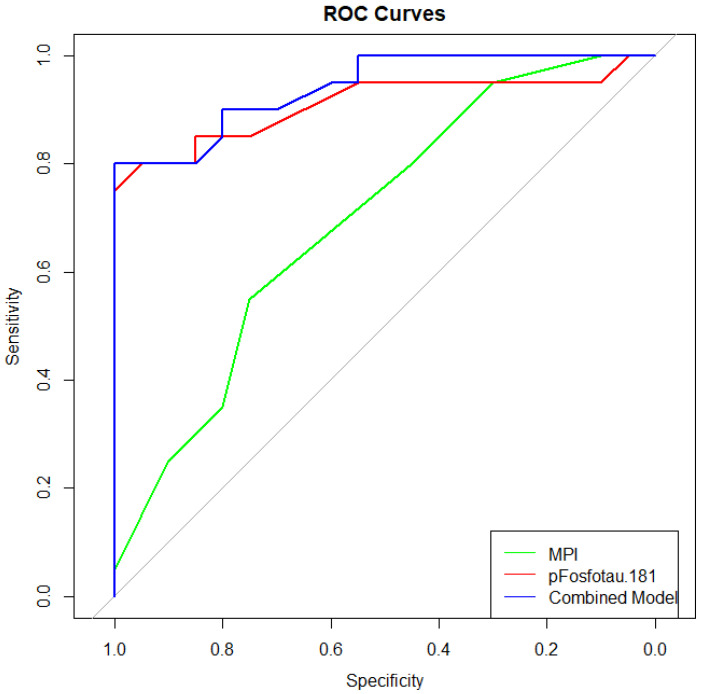
The ROC curves for MPI and p-tau181, separately and in combination, in identifying the AD group.

The diagnostic performance of plasma p-tau181, MPI, and their combination was assessed using ROC curve analysis. Plasma p-tau181 demonstrated the highest discriminative power for AD, with an AUC of 0.910 (95% CI: 0.808–1.00), yielding a sensitivity of 80.0% and specificity of 95.0% at the optimal cut-off. MPI alone showed moderate discrimination, with an AUC of 0.696 (95% CI: 0.534–0.858), sensitivity of 55.0%, and specificity of 75.0%. Finally, the combined model (MPI + p-tau181) significantly improved classification accuracy, achieving an AUC of 0.941 (95% CI: 0.874–1.00), with a sensitivity of 90.0% and specificity of 100% (Figure 1).

Figure 1 illustrates the ROC curves for MPI and p-tau181, separately and in combination, in identifying the AD group.

## 4. Discussion

This study explored the relationship between frailty and AD pathology by combining biological and clinical data. We focused on plasma biomarkers and the MPI, trying to understand how they might be used together in the diagnostic process. Our findings confirm previous evidence on the diagnostic utility of plasma p-tau181 in AD diagnosis, and suggest that frailty, as measured by MPI, may play a relevant role in this context.

Among all the plasma biomarkers we tested, p-tau181 showed significant correlations with CSF p-tau181 and Aβ42/40 ratio, supporting its reliability in identifying AD-related changes, even in a clinical setting. These results are in line with previous research highlighting the value of plasma p-tau181 as a potential non-invasive biomarker for AD, especially when lumbar puncture is not feasible [43].

In logistic regression models adjusted for demographic variables, plasma p-tau181 was significantly associated with AD diagnosis. MPI showed a trend toward significance, and when we combined the two measures, diagnostic accuracy improved. This suggests that functional vulnerability, as captured by MPI, might offer additional information beyond biological markers alone. We also tested a model including an interaction term between plasma p-tau181 and MPI, which showed a marginally significant effect. These findings may indicate that frailty might modulate the underlying biological processes of AD; however, further studies are required to confirm this hypothesis. Some authors have already proposed that frailty could amplify the effects of neurodegeneration or make individuals more sensitive to pathological brain changes [9,10]. Interestingly, we found that MPI was positively associated with plasma p-tau181 levels, even after adjusting for age, sex, and education. This association was not observed for CSF biomarkers [44]. The reasons for this discrepancy are not fully clear, but it may reflect group-specific patterns, since plasma p-tau181 is more closely linked to AD-related amyloid and tau pathology. At the same time, this correlation should not be over-interpreted as a direct pathophysiological link but could rather reflect shared vulnerability or the impact of cognitive impairment, which are both captured by MPI. Further studies are needed to confirm these interpretations.

A more detailed analysis showed that within the MPI domains, the subscales IADL and SPMSQ were those most strongly associated with plasma p-tau181 levels. These associations were present only in the AD group and not in the non-AD group. This may indicate that cognitive frailty—rather than global frailty—is particularly relevant in the context of AD pathology. This observation is consistent with emerging literature on cognitive frailty, which has been described as the co-occurrence of physical frailty and cognitive impairment not fulfilling criteria for dementia. Studies have shown that individuals with cognitive frailty may exhibit a specific subcortical-frontal pattern of dysfunction that is distinct from typical AD [45] and may represent an intermediate stage in the trajectory from healthy aging to dementia. Neuroimaging studies have shown that higher frailty index scores are associated with reduced cortical and thalamic volumes [10], and that only some frailty components, such as sensory impairments and polypharmacy, are closely linked with cortical atrophy. These findings suggest that frailty indices incorporating brain-related domains may be more informative in dementia research. This observation may have practical consequences. If plasma p-tau181 is more closely linked to cognitive-functional vulnerability, then tools like IADL and SPMSQ may help identify patients at higher risk for AD-related pathology. Some authors have proposed that combining cognitive screening tools with biomarker analysis might help improve early diagnosis and clinical decision-making [10,46]. Specifically, easy-to-administer tools that reflect daily functioning may provide ecologically valid insights into disease impact in real-world settings, especially when biomarker testing is not readily available. Recent literature emphasizes the value of integrating functional data into diagnostic frameworks, highlighting that cognitive frailty assessments may not only improve diagnostic sensitivity but also guide prognosis and individualized care planning [47]. While our interpretation focused on AD because MPI scores were more associated with AD-specific biomarkers, we acknowledge that cognitive frailty is a broader construct. Frailty may also contribute to non-AD forms of cognitive decline, and this possibility should be further explored in future studies.

In line with the aims of this work, we intentionally adopted a multidomain definition of frailty. The MPI was chosen because it encompasses not only physical but also cognitive, functional, nutritional, and social dimensions, thus reflecting the real-world complexity of dementia. While this multidimensionality may partly overlap with disease severity, it provides a comprehensive framework to capture patient vulnerability. In this sense, MPI complements biomarker information by adding clinically meaningful context that purely physical frailty indices cannot provide.

Another relevant finding is the improvement in diagnostic accuracy when MPI is used together with plasma p-tau181. ROC curve analysis showed that the combination reached excellent performance (AUC = 0.941) and was able to correctly identify all AD cases in our sample. This suggests that using both biological and functional measures could be a promising strategy for improving the diagnostic process, especially in clinical settings where time and resources are limited. From a clinical perspective, both plasma biomarkers and MPI are non-invasive, repeatable over time, and relatively easy to apply. Their combined use might allow clinicians to better understand patient vulnerability and tailor management strategies accordingly. Although MPI on its own had limited discriminatory power (AUC = 0.696), its combination with plasma p-tau181 improved diagnostic performance. This indicates that frailty assessment may provide additional context and support biomarker-based classification, rather than serving as an independent diagnostic tool.

Despite these encouraging results, our study has several limitations. The relatively small sample size (n = 40) limits statistical power and may increase the risk of overfitting. Therefore, our findings should be interpreted with caution and confirmed in larger, longitudinal cohorts. Furthermore, we did not include in our analysis p-tau217, despite its high diagnostic potential for AD reported in the recent literature. Also, the cross-sectional design limits our ability to explore causal relationships or long-term outcomes. Finally, although MPI has been validated as a prognostic tool, its use in the diagnostic phase of cognitive disorders remains experimental. Our findings support the idea that frailty assessment could contribute to early diagnosis, especially when combined with biomarkers, but this needs to be confirmed in larger, longitudinal studies.

## 5. Conclusions

Our study suggests that integrating functional frailty measures with plasma biomarkers enhances the accuracy and interpretability of Alzheimer’s disease diagnosis. Frailty, operationalized through the MPI, showed modest but suggestive associations with biomarker profiles. It could provide complementary clinical information when considered together with biological markers. This combined perspective could represent a useful direction for future research and diagnostic practices in dementia.

Plasma p-tau181 confirms its role as a core blood-based biomarker for AD, while MPI offers clinically meaningful context regarding patient resilience and functional reserve. Together, they represent a feasible, scalable approach for early diagnosis and risk stratification. In this study our goal improves the diagnostic role of p-tau181, providing a complementary clinical perspective using MPI as a functional index. Future studies with larger cohorts and longitudinal designs should explore whether combining these tools can improve prognostication, personalize treatment, and guide care planning in patients with cognitive impairment.

## Figures and Tables

**Table 1:** Demographic characteristics, biomarkers and neuropsychological findings of AD and non-AD patients. Gender is expressed as percentages (%). Continuous variables with normal distribution are reported as mean ± standard deviation (SD). Ordinal variables (MPI, ADL, IADL, SPMSQ, ESS, MNA, CIRS-CI, number of drugs, social condition) are reported as median (interquartile range, IQR). Group comparisons were assessed using appropriate statistical tests, and the corresponding *p*-values are shown in the rightmost column.

	Non-AD	AD	*p*-Value
Age	66.2 ± 9.60	69.8 ± 8.21	0.139
Sex	7 (35%)	10 (50%)	0.343
Education	11.7 ± 3.39	11.3 ± 3.50	0.569
CSF Aβ42	722 ± 312	340 ± 149	<0.001
CSF Aβ42/40 ratio	0.0794 ± 0.0146	0.0417 ± 0.0116	<0.001
CSF Tau	370 ± 149	644 ± 308	0.001
CSF p-tau181	45.3 ± 29.3	113 ± 58	<0.001
LCS NfL	2269 ± 2906	1107 ± 704	0.417
plasma Aβ42	27.9 ± 7.41	22.8 ± 8.81	0.026
plasma Aβ42/40 ratio	0.0925 ± 0.0133	0.0765 ± 0.0107	<0.001
plasma p-tau181	0.927 ± 0.294	2.33 ± 1.15	<0.001
plasma NfL	40.6 ± 35.6	30.5 ± 15.2	0.626
MPI	0.188, 0.0625–0.203	0.250, 0.188–0.328	0.031
ADL	6, 6–6	6, 6–6	0.668
IADL	8, 7–8	7, 4.75–8	0.090
ESS	20, 19,8–20	20, 19,8–20	0.921
SPMSQ	1, 1–2	2, 1–5	0.021
MNA	12, 11–14	12, 11–14	0.687
CIRS-CI	2, 0.75–2.25	2.50, 1–3	0.589
NUMBER OF DRUGS	2, 2–3	2.50, 2–5.25	0.205
SOCIAL CONDITION	0, 0–1	0, 0–0.5	0.478
MMSE	25.7 ± 4.71	21.6 ± 7.61	0.027
MoCA	19.4 ± 4.83	16.8 ± 5.70	0.204
FAB	13.1 ± 3.80	11.8 ± 3.56	0.269
CLOCK	10.6 ± 3.08	9.35 ± 4.53	0.420

## Data Availability

The data presented in this study are not publicly available due to privacy and ethical restrictions.

## Abbreviations

The following abbreviations are used in this manuscript:

AD	Alzheimer’s Disease
MPI	Multidimensional Prognostic Index
Aβ42	Amyloid Beta 1–42
Aβ40	Amyloid Beta 1–40
p-tau181	Phosphorylated Tau 181
tTau	Total Tau
NfL	Neurofilament light
CSF	Cerebrospinal Fluid
MMSE	Mini-Mental State Examination
MoCA	Montreal Cognitive Assessment
FAB	Frontal Assessment Battery
CDT	Clock Drawing Test
SPMSQ	Short Portable Mental Status Questionnaire
ESS	Exton Smith Scale
MNA	Mini Nutritional Assessment
CIRS	Cumulative Illness Rating Scale
ADL	Activities of Daily Living
IADL	Instrumental Activities of Daily Living
FTD	Frontotemporal Dementia
DLB	Dementia with Lewy Bodies
VD	Vascular Dementia
ROC	Receiver Operating Characteristic
AUC	Area Under the Curve
CLEIA	Chemiluminescent Enzyme Immunoassay
APOE	Apolipoprotein E
PET	Positron Emission Tomography

## References

[1] Corral-Pérez J., Casals C., Ávila-Cabeza-de-Vaca L., González-Mariscal A., Martínez-Zaragoza I., Villa-Estrada F., Reina-Campos R., Vázquez-Sánchez M.Á. (2023). Health factors associated with cognitive frailty in older adults living in the community. Front. Aging Neurosci..

[2] Clegg A., Bates C., Young J., Ryan R., Nichols L., Teale E.A., Mohammed M.A., Parry J., Marshall T. (2016). Development and validation of an electronic frailty index using routine primary care electronic health record data. Age Ageing.

[3] Fried L.P., Tangen C.M., Walston J., Newman A.B., Hirsch C., Gottdiener J., Seeman T., Tracy R., Kop W.J., Burke G. (2001). Frailty in older adults: Evidence for a phenotype. J. Gerontol. A Biol. Sci. Med. Sci..

[4] Wang S., Li Q., Wang S., Huang C., Xue Q.-L., Szanton S.L., Liu M. (2024). Sustained Frailty Remission and Dementia Risk in Older Adults: A Longitudinal Study. Alzheimer’s Dement..

[5] Franceschi C., Campisi J. (2014). Chronic inflammation (inflammaging) and its potential contribution to age-associated diseases. J. Gerontol. A Biol. Sci. Med. Sci..

[6] Soysal P., Stubbs B., Lucato P., Luchini C., Solmi M., Peluso R., Sergi G., Isik A.T., Manzato E., Maggi S. (2016). Inflammation and frailty in the elderly: A systematic review and meta-analysis. Ageing Res. Rev..

[7] Zhou Y., Zhao Y., Li Y., Hu N., Gong L., Zhao Q., Guo Q. (2022). Association of Frailty with Plasma Phosphorylated Tau in Community-Dwelling Older Adults. BMC Geriatr..

[8] Wallace L.M.K., Theou O., Pena F., Rockwood K. (2021). Frailty Trajectories in Three Cohorts of Community-Dwelling Older Adults: A Ten-Year Follow-Up Study. Aging Med..

[9] Kocagoncu E., Nesbitt D., Emery T., Hughes L.E., Henson R.N., Rowe J.B., Cambridge Centre for Ageing and Neuroscience (2022). Neurophysiological and brain structural markers of cognitive frailty differ from Alzheimer’s disease. J. Neurosci..

[10] Gutiérrez-Zúñiga R., Davis J.R.C., Ruddy K., De Looze C., Carey D., Meaney J., Kenny R.A., Knight S.P., Romero-Ortuno R. (2023). Structural brain signatures of frailty, defined as accumulation of self-reported health deficits in older adults. Front. Aging Neurosci..

[11] Canevelli M., Jackson-Tarlton C., Rockwood K. (2024). Frailty for neurologists: Perspectives on how frailty influences care planning. Lancet Neurol..

[12] Scheltens P., De Strooper B., Kivipelto M., Holstege H., Chételat G., Teunissen C.E., Cummings J., Van Der Flier W.M. (2021). Alzheimer’s disease. Lancet.

[13] Jack C.R., Bennett D.A., Blennow K., Carrillo M.C., Dunn B., Haeberlein S.B., Holtzman D.M., Jagust W., Jessen F., Karlawish J. (2018). NIA-AA Research Framework: Toward a biological definition of Alzheimer’s disease. Alzheimers Demen..

[14] Jack C.R., Jr Andrews J.S., Beach T.G., Buracchio T., Dunn B., Graf A., Hansson O., Ho C., Jagust W., McDade E. (2024). Revised criteria for diagnosis and staging of Alzheimer’s disease: Alzheimer’s Association Workgroup. Alzheimers Demen..

[15] Frisoni G.B., Festari C., Massa F., Cotta Ramusino M., Orini S., Aarsland D., Agosta F., Babiloni C., Borroni B., Cappa S.F. (2024). European intersocietal recommendations for the biomarker-based diagnosis of neurocognitive disorders. Lancet Neurol..

[16] Beauchet O., Matskiv J., Gaudreau P., Allali G., Vaillant-Ciszewicz A.-J., Guerin O., Grose A. (2023). Frailty, Cognitive Impairment, and Incident Major Neurocognitive Disorders: Results of the NuAge Cohort Study. J. Alzheimers Dis..

[17] Ward D.D., Ranson J.M., Wallace L.M.K., Llewellyn D.J., Rockwood K. (2022). Frailty, lifestyle, genetics and dementia risk. J. Neurol. Neurosurg. Psychiatry.

[18] Cruz-Jentoft A.J., Daragjati J., Fratiglioni L., Maggi S., Mangoni A.A., Mattace-Raso F., Paccalin M., Polidori M.C., Topinkova E., Ferrucci L. (2020). Using the Multidimensional Prognostic Index (MPI) to improve cost-effectiveness of interventions in multimorbid frail older persons: Results and final recommendations from the MPI_AGE European Project. Aging Clin. Exp. Res..

[19] Overbeek F.C.M.S., Goudzwaard J.A., van Hemmen J., van Bruchem-Visser R.L., Papma J.M., Polinder-Bos H.A., Mattace-Raso F.U.S. (2022). The Multidimensional Prognostic Index Predicts Mortality in Older Outpatients with Cognitive Decline. J. Clin. Med..

[20] Pilotto A., Ferrucci L., Franceschi M., D’Ambrosio L.P., Scarcelli C., Cascavilla L., Paris F., Placentino G., Seripa D., Dallapiccola B. (2008). Development and validation of a multidimensional prognostic index for one-year mortality from comprehensive geriatric assessment in hospitalized older patients. Rejuvenation Res..

[21] Veronese N., Custodero C., Cella A., Demurtas J., Zora S., Maggi S., Barbagallo M., Sabbà C., Ferrucci L., Pilotto A. (2021). Prevalence of multidimensional frailty and pre-frailty in older people in different settings: A systematic review and meta-analysis. Ageing Res. Rev..

[22] Buscarnera S., Canevelli M., Bruno G., Garibotto V., Frisoni G.B., Ribaldi F. (2025). Unraveling the link between frailty and Alzheimer’s disease biomarkers in patients with mild cognitive impairment. GeroScience.

[23] Sourdet S., Soriano G., Delrieu J., Steinmeyer Z., Guyonnet S., Saint-Aubert L., Payoux P., Ousset P.J., Ghisolfi A., Chicoulaa B. (2021). Cognitive Function and Amyloid Marker in Frail Older Adults: The COGFRAIL Cohort Study. J. Frailty Aging.

[24] Ngandu T., Lehtisalo J., Solomon A., Levälahti E., Ahtiluoto S., Antikainen R., Bäckman L., Hänninen T., Jula A., Laatikainen T. (2015). A 2 Year Multidomain Intervention of Diet, Exercise, Cognitive Training, and Vascular Risk Monitoring versus Control to Prevent Cognitive Decline in At-Risk Elderly People (FINGER): A Randomised Controlled Trial. Lancet.

[25] Rascovsky K., Hodges J.R., Knopman D., Mendez M.F., Kramer J.H., Neuhaus J., van Swieten J.C., Seelaar H., Dopper E.G., Onyike C.U. (2011). Sensitivity of revised diagnostic criteria for the behavioural variant of frontotemporal dementia. Brain.

[26] McKeith I.G., Boeve B.F., Dickson D.W., Halliday G., Taylor J.P., Weintraub D., Aarsland D., Galvin J., Attems J., Ballard C.G. (2017). Diagnosis and management of dementia with Lewy bodies: Fourth consensus report of the DLB Consortium. Neurology.

[27] Sachdev P., Kalaria R., O’Brien J., Skoog I., Alladi S., Black S.E., Blacker D., Blazer D.G., Chen C., Chui H. (2014). Diagnostic criteria for vascular cognitive disorders: A VASCOG statement. Alzheimer Dis. Assoc. Disord..

[28] Wilson S.M., Galantucci S., Tartaglia M.C., Gorno-Tempini M.L. (2012). The neural basis of syntactic deficits in primary progressive aphasia. Brain Lang..

[29] Hansson O., Batrla R., Brix B., Carrillo M.C., Corradini V., Edelmayer R.M., Esquivel R.N., Hall C., Lawson J., Bastard N.L. (2021). The Alzheimer’s Association international guidelines for handling of cerebrospinal fluid for routine clinical measurements of amyloid β and tau. Alzheimers Dement..

[30] O’Bryant S.E., Gupta V., Henriksen K., Edwards M., Jeromin A., Lista S., Bazenet C., Soares H., Lovestone S., Hampel H. (2015). Guidelines for the standardization of preanalytic variables for blood-based biomarker studies in Alzheimer’s disease research. Alzheimers Dement..

[31] Roveta F., Rubino E., Marcinnò A., Grassini A., Piella E.M., Ferrandes F., Bonino L., Giaracuni G., Boschi S., Gioiello G. (2025). Diagnostic performance of plasma biomarkers for Alzheimer’s disease using a fully automated platform: A real-world clinical study. J. Alzheimers Dis..

[32] Folstein M.F., Robins L.N., Helzer J.E. (1983). The Mini-Mental State Examination. Arch. Gen. Psychiatry.

[33] Nasreddine Z.S., Phillips N.A., Bédirian V., Charbonneau S., Whitehead V., Collin I., Cummings J.L., Chertkow H. (2005). The Montreal Cognitive Assessment, MoCA: A brief screening tool for mild cognitive impairment. J. Am. Geriatr. Soc..

[34] Sunderland T., Hill J.L., Mellow A.M., Lawlor B.A., Gundersheimer J., Newhouse P.A., Grafman J.H. (1989). Clock drawing in Alzheimer’s disease. A novel measure of dementia severity. J. Am. Geriatr. Soc..

[35] Dubois B., Slachevsky A., Litvan I., Pillon B. (2000). The FAB: A Frontal Assessment Battery at bedside. Neurology.

[36] Katz S., Ford A.B., Moskowitz R.W., Jackson B.A., Jaffe M.W. (1963). studies of illness in the aged. the index of ADL: A standardized measure of biological and psychosocial function. JAMA.

[37] Lawton M.P., Brody E.M. (1969). Assessment of older people: Self-maintaining and instrumental activities of daily living. Gerontologist.

[38] Pfeiffer E. (1975). A short portable mental status questionnaire for the assessment of organic brain deficit in elderly patients. J. Am. Geriatr. Soc..

[39] Exton-Smith A.N., Sherwin R.W. (1961). The prevention of pressure sores. Significance of spontaneous bodily movements. Lancet.

[40] Vellas B., Guigoz Y., Garry P.J., Nourhashemi F., Bennahum D., Lauque S., Albarede J.L. (1999). The Mini Nutritional Assessment (MNA) and its use in grading the nutritional state of elderly patients. Nutrition.

[41] Linn B.S., Linn M.W., Gurel L. (1968). Cumulative illness rating scale. J. Am. Geriatr. Soc..

[42] Pilotto A., Sancarlo D., Pellegrini F., Rengo F., Marchionni N., Volpato S., Ferrucci L., FIRI-SIGG Study Group (2016). The Multidimensional Prognostic Index predicts in-hospital length of stay in older patients: A multicentre prospective study. Age Ageing.

[43] González-Escalante A., Milà-Alomà M., Brum W.S., Ashton N.J., Ortiz-Romero P., Shekari M., Campo M.D., Anastasi F., Quijano-Rubio C., Kollmorgen G. (2025). A plasma biomarker panel for detecting early amyloid-β accumulation and its changes in middle-aged cognitively unimpaired individuals at risk for Alzheimer’s disease. EBioMedicine.

[44] Zhang X.M., Wu X.J., Cao J., Jiao J., Chen W. (2022). Association between Cognitive Frailty and Adverse Outcomes among Older Adults: A Meta-Analysis. J. Nutr. Health Aging.

[45] Sabbagh M.N., Lue L.F., Fayard D., Shi J. (2017). Increasing Precision of Clinical Diagnosis of Alzheimer’s Disease Using a Combined Algorithm Incorporating Clinical and Novel Biomarker Data. Neurol. Ther..

[46] Nader M.M., Cosarderelioglu C., Miao E., Whitson H., Xue Q.L., Grodstein F., Oh E., Ferrucci L., Bennett D.A., Walston J.D. (2023). Navigating and Diagnosing Cognitive Frailty in Research and Clinical Domains. Nat. Aging.

[47] Wallace L., Theou O., Rockwood K., Andrew M.K. (2018). Relationship between Frailty and Alzheimer’s Disease Biomarkers: A Scoping Review. Alzheimers Dement..

